# Membrane Distillation: Recent Configurations, Membrane Surface Engineering, and Applications

**DOI:** 10.3390/membranes11120934

**Published:** 2021-11-26

**Authors:** Sundararajan Parani, Oluwatobi Samuel Oluwafemi

**Affiliations:** 1 Department of Chemical Sciences, University of Johannesburg, Doornfontein, Johannesburg 2028, South Africa; parani.sundararajan@gmail.com; 2 Center for Nanomaterials Science Research, University of Johannesburg, Doornfontein, Johannesburg 2028, South Africa

**Keywords:** membrane distillation, membrane materials, membrane configurations, surface engineering, water treatment

## Abstract

Membrane distillation (MD) is a developing membrane separation technology for water treatment that involves a vapor transport driven by the vapor pressure gradient across the hydrophobic membrane. MD has gained wide attention in the last decade for various separation applications, including the separation of salts, toxic heavy metals, oil, and organic compounds from aqueous solutions. Compared with other conventional separation technologies such as reverse osmosis, nanofiltration, or thermal distillation, MD is very attractive due to mild operating conditions such as low temperature and atmospheric pressure, and 100% theoretical salt rejection. In this review, membrane distillation’s principles, recent MD configurations with their advantages and limitations, membrane materials, fabrication of membranes, and their surface engineering for enhanced hydrophobicity are reviewed. Moreover, different types of membrane fouling and their control methods are discussed. The various applications of standalone MD and hybrid MD configurations reported in the literature are detailed. Furthermore, studies on the MD-based pilot plants installed around the world are covered. The review also highlights challenges in MD performance and future directions.

## 1. Introduction

Life on Earth depends on water for survival. Nearly 70% of the earth is covered by water. Of this, only 2.5% is freshwater, and the rest is ocean based. However, only 1% of the freshwater is readily accessible, which is found in surface water such as lakes, swamps, rivers, and streams, while the rest of it is trapped as ice in glaciers and snowfields. The current era faces a major challenge of freshwater scarcity across the globe due to growing population, contamination of water sources, industrial pollution, climate change, excessive and misuse of groundwater, and accordingly, the demand for freshwater against supply has increased dramatically. There are reports that nearly 785 million people, which is ~10% of the world population, currently have no access to clean freshwater, and this is projected to affect 1.8 billion by 2025 [1]. As a result, this has led to the use of different strategies to extract freshwater from seawater and brackish water via desalination to meet the demand. The current desalination plants are majorly based on thermal and membrane processes. The thermal processes such as multistage flash (MSF) and multi-effect distillation (MED) accounts for 25% global desalination capacity while reverse osmosis (RO) membrane technology, being the largest contributor, contributes about 69% [2]. These desalination techniques operate at high temperatures or consume high electrical energy, which are currently fulfilled by fossil fuels such as coal, petroleum, etc. As these fossil fuels are non-sustainable and rapidly depleting, an alternative desalination technique with a minimum energy requirement involving sustainable energy sources is needed for freshwater production.

## 2. Membrane Distillation

**Figure 1:**
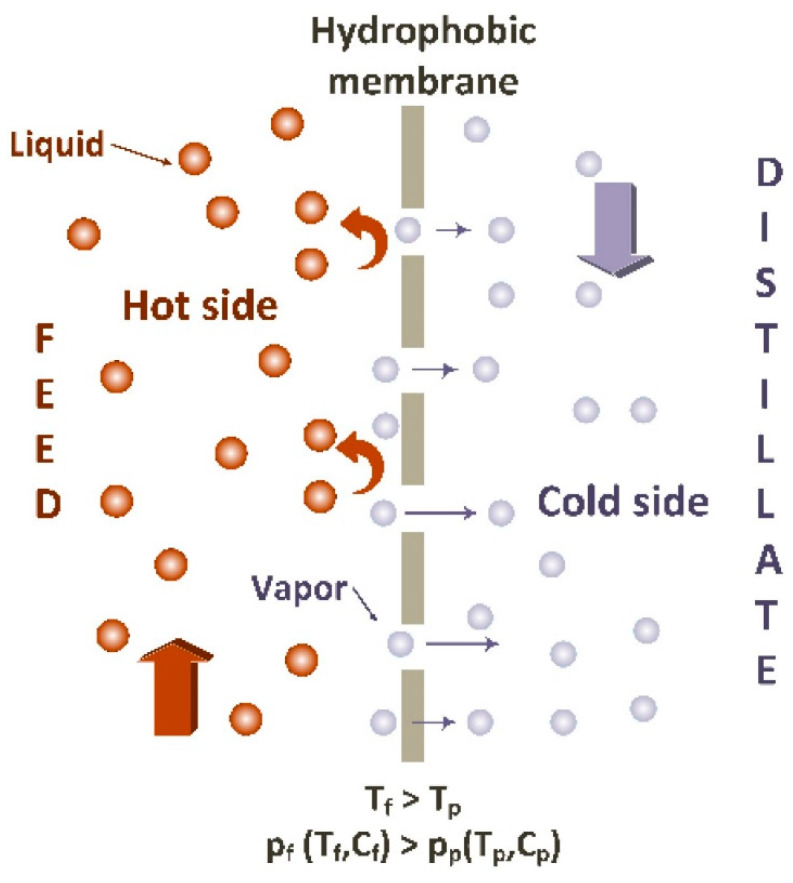
Membrane distillation (MD) principles [5].

In recent years, the membrane distillation (MD) technique has gained substantial attention for desalination and wastewater treatment [3,4]. MD is a thermally driven process that uses the microporous hydrophobic membrane to separate the vapor phase from the liquid phase. In MD, the non-volatile components of the aqueous feed solution are retained in the feed solution by hydrophobic surface while the volatile parts (water vapor) are transported through the porous structure and collected as a distillate in a pure form (Figure 1). The driving force for the vapor transfer is the vapor pressure difference (ΔP) induced by temperature difference (ΔT) across the membrane [5]. This enables the separation of non-volatile solutes from water at lower operating temperatures and makes it possible to use low-grade waste heat and/or sustainable solar energy as heat sources that are widely available. Unlike RO, MD operates at atmospheric pressure but exhibits high ions and non-volatile rejections up to 99.9% from the solution, even close to saturated concentration [6,7]. Owing to the number of such advantages, MD appears to be cost-effective and energy-efficient with wide applications in desalination.

## 3. Conventional Configurations

**Figure 2:**
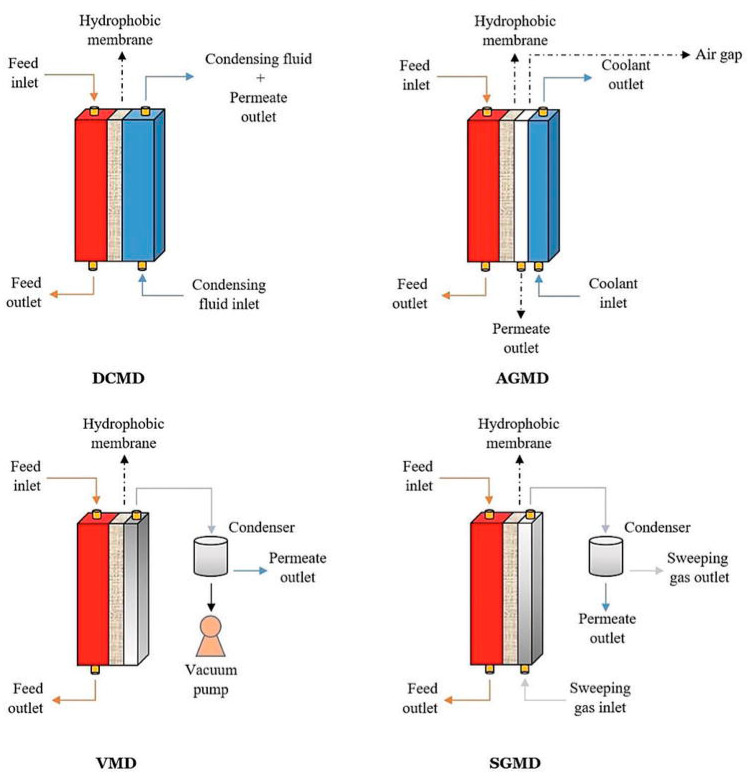
Conventional configurations of membrane distillation [10].

MD processes can be majorly classified into four major configurations depending on the methods to induce a vapor pressure difference across the membrane and the type of the permeate collection on the cold side [8,9,10]. It is mandatory that the feed solution is in contact with the hydrophobic surface of the membrane in all configurations. Figure 2 illustrates the conventional configurations of the membrane distillation system and Table 1 presents their advantages, limitations, and applications.

### 3.1. Direct Contact Membrane Distillation (DCMD)

DCMD is the simplest and oldest configuration proposed where both feed and permeate solutions are in direct contact with the membrane. The feed solution is heated and circulated on the hydrophobic surface of the membrane while the cold permeate is circulated on the other side of the membrane. The water is vaporized at the membrane surface which is then transported to the membrane pores and condensed on the permeate side. As mentioned earlier, the vapor pressure difference caused by the temperature difference on the two sides of the membrane is the driving force for this mass transfer. The main disadvantage of this method is the conductive heat loss during the operation, which reduces the thermal and pressure gradient across the membrane. DCMD has been the most investigated configuration in the desalination of seawater, brackish water, and hypersaline solution.

### 3.2. Air-Gap Membrane Distillation (AGMD)

In a typical AGMD process, the hot feed solution is in direct contact with the hydrophobic surface of the membrane while a thin stagnant airgap is introduced between the permeate side of the membrane and a condensation surface. The thickness of the airgap is usually between 2–10 mm. The vapor passes through the membrane and the air gap and condenses on the cold surface (condensing plate) rather than directly in the cold liquid as in DCMD. As seen in Figure 2, the permeate channel is separated from the cold stream. This design could reduce the conductive heat loss across the membrane. However, the permeate flux obtained is usually low with this design since the water vapor has to transmit through the air barrier, which acts as additional resistance to the mass transfer. Partial vacuum can also be applied in the airgap to extract, and such configuration is called vacuum assisted AGMD (V-AGMD). This configuration is designed to remove the non-condensable gases in the feed solution that causes additional vapor transfer resistance [11,12]. AGMD is advantageous over DCMD in the separation of high volatile organic compounds from water as the permeate is not in contact with the distillate surface of the membrane and hence the chances of membrane wetting are low.

### 3.3. Vacuum Membrane Distillation (VMD)

In VMD configuration, vacuum is applied at the permeate side of the membrane to collect the vapors, and the vapor condensation takes place outside the membrane module. The vacuum pressure is maintained less than the saturation pressure of the volatile solvent to be collected from the feed solution. Compared to DCMD and AGMD, VMD exhibits negligible conductive heat loss and high permeate flux. However, in VMD, the membrane is susceptible to wetting severely due to the applied vacuum pressure. Like AGMD, VMD is useful to remove volatile organic compounds (VOC) from the feed solution.

### 3.4. Sweeping Gas Membrane Distillation (SGMD)

In SGMD, cold, inert gas is passed through the permeate side of the membrane to sweep the vapors, which are then condensed outside the membrane module. This configuration has a gas barrier to reduce the conductive heat loss like in AGMD, to increase the mass transfer. The major shortcoming of this configuration is that it requires a big condenser because of the large volume of the sweep gas. SGMD is the least studied configuration, and it is useful for removing volatile organics from the feed solution.

## 4. Recent Configurations

### 4.1. Permeate Gap Membrane Distillation (PGMD)

**Figure 3:**
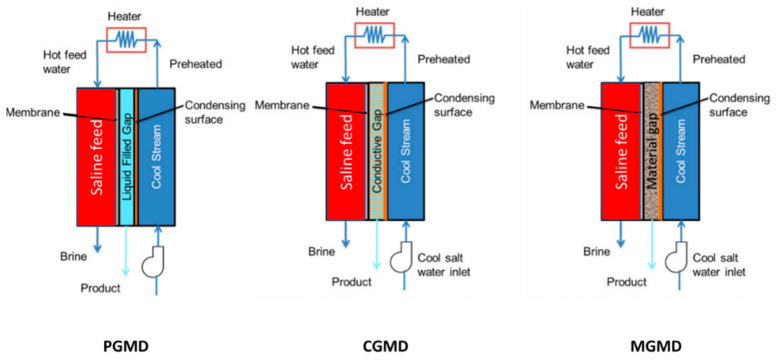
PGMD, CGMD, and MGMD configurations. Adapted from [14]. Copyright (2016) with permission from Elsevier.

PGMD is the current configuration developed by combining the airgap and direct contact membrane distillation modules. In PGMD mode, the air gap in the AGMD is filled by permeate water (Figure 3). As in DCMD, the vapor condenses immediately after leaving the membrane, which is then condensed by the coolant. As permeate water does not mix with the coolant, the coolant can be any liquid, even the cold-feed water. In this case, cold feed solution is first pumped to a cold stream, then heated outside the cold stream, and passed through a hot stream. Compared to AGMD, PGMD exhibits lower mass transfer resistance for vapor transport due to the introduction of permeate water in the gap. Compared to DCMD, PGMD also exhibits thermal conductivity across the membrane. However, this thermal energy will be carried out to preheat the cold stream, thus minimizing the energy consumption for the hot feed solution. In addition, PGMD does not require precooled permeate, whereas, in DCMD, it is needed.

### 4.2. Material Gap Membrane Distillation (MGMD)

In further advancements, the air gap in AGMD is filled by different materials, which can be classified as thermally conducting or non-conducting materials (Figure 3). This includes metal mesh polypropylene mesh, polyurethane sponge, sand, etc. [13]. These attempts are made to increase the permeate flux and/or decrease the conductive heat loss across the membrane. The configuration that uses the conducting plate in the gap is also called conducting gap membrane distillation (CGMD), and higher heat loss is expected similar to DCMD, but it could also help to reject the non-volatile compounds at low temperature.

### 4.3. Feed Gap Membrane Distillation (FGMD)

**Figure 4:**
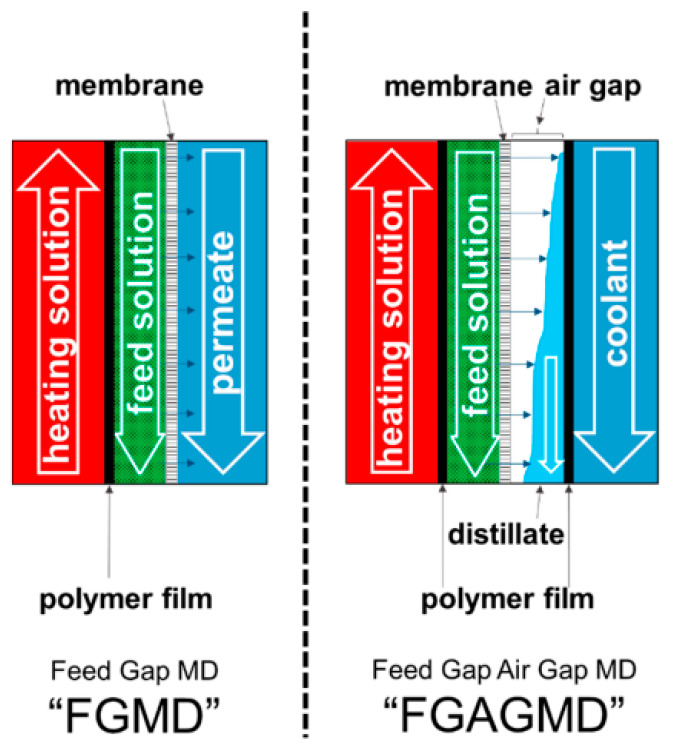
FGMD and FGAGMD configurations [15].

The FGMD mode is the horizontally flipped arrangement of AGMD mode where the feed solution is circulated in the air gap which is heated by the heating solution introduced in the former coolant channel of AGMD (Figure 4). The permeate channel is the former feed channel in AGMD. The heating solution and the feed channel is separated by a heating plate or film. This configuration is to increase the permeate output, which is limited by sensible heat contained in permeate stream. Advancement to this is the feed gap and air gap membrane distillation (FGAGMD) configuration. Where an air gap is maintained between feed and permeate, and the latter will be externally cooled by the coolant as in AGMD. Accordingly, in FGAGMD, both the feed and permeate are separated from their heating and cooling solution. This FGAGMD configuration is useful for the treatment of industrial wastewater containing corrosive and toxic substances [15].

## 5. Membrane Materials and Fabrication

Since hydrophobicity is the basic requirement for membrane distillation process, the membrane material must be intrinsically hydrophobic, or its surface must be modified to be hydrophobic. The most used membranes for MD are polypropylene (PP), polytetrafluoroethylene (PTFE), and polyvinylidene fluoride (PVDF) [16]. Among these, PTFE has advantages such as high chemical stability, high crystallinity, low intermolecular attraction, low surface energy, and high hydrophobicity [17]. However, the major challenge with PTFE is its insolubility in any solvent. PTFE membrane is usually fabricated by the sintering-extrusion or melt-extrusion process [18,19]. In the sintering process, ultrafine powder of PTFE is mixed with the volatile lubricating agent to form the paste, which is then extruded into a flat sheet or hollow fiber. This is later heated and stretched in order to produce membranes with microporous nature. Alternatively, in the melt-extrusion method, microporous PTFE membranes are produced by melting the PTFE powders, followed by extrusion and stretching. However, both of these physical processes are expensive. In addition, the chemical synthesis of PTFE powders is more complex, time-consuming, and also involves the release of harmful substances into the environment [20].

PP is a low-cost crystalline polymer with good thermal and mechanical properties, and good chemical stability, thus suitable for membrane applications. However, compared with PVDF, PP has high surface energy and hence low hydrophobicity. Porous PP membrane can be prepared by the melt-extrusion stretching method as described above [21]. In addition, the thermally induced phase separation (TIPS) method is also used to prepare PP membrane. In TIPS, PP powders are heated at elevated temperatures in the presence of diluent to form a homogeneous solution which is then allowed to cool or quench to induce phase separation. This is followed by extraction of the diluent in order to obtain a porous membrane [22,23,24].

**Figure 5:**
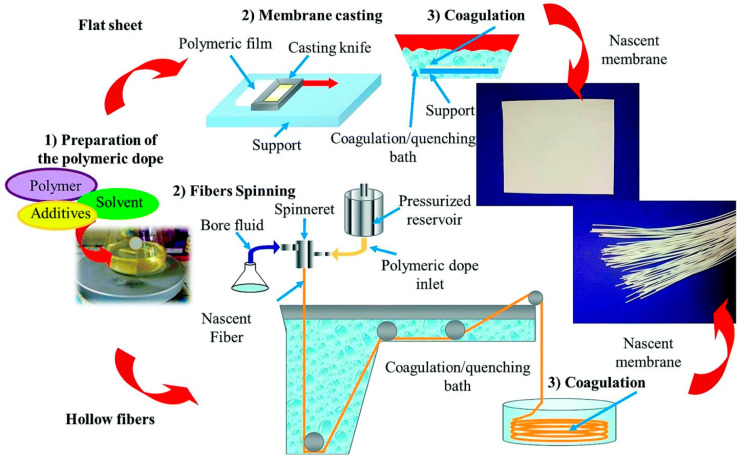
Schematic diagram of the NIPS method to produce flat sheet and hollow fiber membranes. Reproduced from [26], with permission from The Royal Society of Chemistry.

PVDF is a semi-crystalline polymer with good chemical stability, moderate thermal and mechanical properties, and similar surface energy to PP. However, compared to PTFE and PP, PVDF is easily soluble in most organic solvents such as n-methyl-2-pyrrolidone (NMP), dimethylacetamide (DMAC), and dimethylformamide (DMF), thus making membrane preparation very easy. Because of this ease of processability, PVDF has been extensively investigated for membrane distillation. Porous PVDF membranes can be prepared by phase separation methods such as TIPS, as mentioned earlier, non-solvent induced phase separation (NIPS) [25], and vapor-induced phase separation (VIPS). Among them, NIPS is the most investigated method for producing membranes for MD applications. A schematic diagram of NIPS is shown in Figure 5. In a conventional NIPS method, PVDF is dissolved in a solvent or mixture of solvents along with additives followed by stirring/shaking/gentle heating to form a homogeneous solution called a dope solution. The solution is then cast on the support (glass, non-woven polyester etc.) for a flat sheet or passed to the spinneret for a hollow fiber and fabricated as a membrane by immersing into the non-solvent (often water) bath, which acts as the phase-separating medium. This results in the exchange of solvent by nonsolvent and forms a microporous membrane.

**Figure 6:**
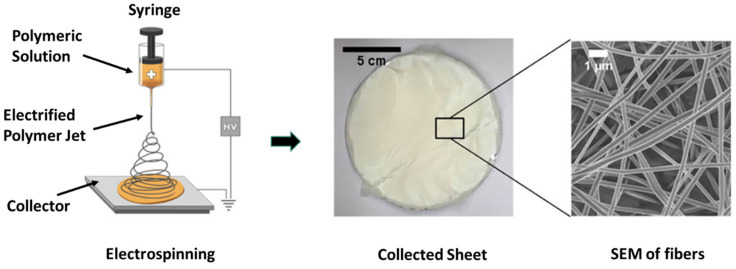
Schematic diagram of electrospinning technique. Modified from [30]: Designing Solutions for Electrospinning of Poly(ionic liquid)s. Macromolecules, 52, 5223–5230. Copyright (2019) with permission from American Chemical Society. Further permissions related to the material excerpted should be directed to the ACS.

In the VIPS method, a non-solvent vapor is passed to the dope solution before it is immersed into a non-solvent bath [27,28]. Electrospinning, an electrostatic-driven process, is also largely used to produce PVDF membrane [29,30]. In the electrospinning method, polymer dope solution is pushed and electrically charged with a high voltage and is ejected through a fine tip as fiber. The electrospun fiber is then collected on the substrate of the collector which is grounded to the earth (Figure 6). The solvent in the fiber is then evaporated and subjected to heat-press treatment to compress all the fibers together in order to form an electrospun membrane. The applied voltage and the distance between the tip and the collector can be varied to obtain the uniform fibre with the desired thickness. The collector can be a stationary or a rotating disk. The advantages of electrospinning include its versatility to produce sub-micron and nanosized fibers with tailored morphology.

Polysulfone (PSf) and polyethersulfone (PES) belong to the polysulfone family and are widely employed commercial membrane materials to produce ultrafiltration membranes and reverse osmosis membranes. PSf and PES can also be considered for membrane distillation because polysulfones are cost-effective and exhibit good mechanical strength. Polysulfone membranes are prepared by phase separation and electrospinning methods. Compared with other polymers, the low thermal conductivity of PES is beneficial to diminish the conductive heat loss and thus improve the heat efficiency of the MD process. Furthermore, its superior rigidity against membrane contraction makes it attractive because membrane contraction is problematic in MD industrialization. The main disadvantage of polysulfone membranes is their hydrophilicity; however, membrane surfaces can be modified to exhibit hydrophobicity for MD applications [31,32,33].

Apart from the aforementioned polymers, polyamide [34], polyacrlyo nitrile [35], polydopamine [36], polyethylene [37], polyetherimide [38], polyethylene terephthalate [39], and so on have been investigated for the preparation of the membrane for MD applications. As most of these polymers produce hydrophilic membranes, their surface requires hydrophobic modifications prior to their applications.

Ceramic membranes have also received attention for MD applications. They are porous inorganic membranes produced by sintering conventional ceramic materials such as alumina, silica, and zirconia. As the ceramics are hydrophilic in nature, a surface modification to produce/enhance hydrophobicity is necessary for them to be suitable for MD operations [40,41,42]. Compared to polymer membranes, ceramic membranes offer high mechanical strength, however, they are expensive due to high temperature (>1000 °C) requirements for the sintering process.

## 6. Surface Engineering

The frequent problem associated with the membrane is ‘pore wetting’ and fouling. During continuous operation, the membrane surface is susceptible to interaction with reactive components of the feed solution, thus making the membrane hydrophilic with its pores wetted by feed solution. This will greatly affect the separation efficiency as the feed water could enter the membrane. In addition, there is a greater possibility for fouling due to the accumulation of unwanted species such as salt, particulates, or biofilms on the membrane surface and pores [43]. This could result in vapor transfer or in pore wetting.

**Figure 7:**
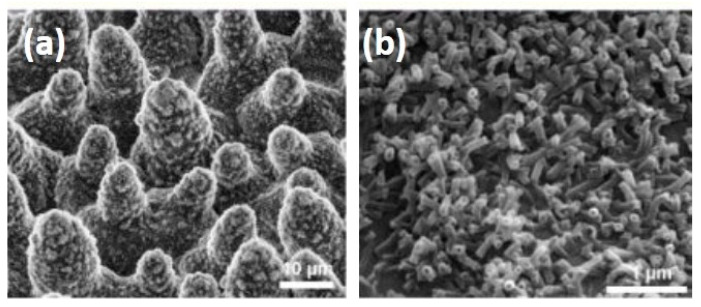
(**a**) Lower and (**b**) higher magnification SEM image of lotus leaf showing the hierarchical surface structure consisting of papillae, wax clusters, and wax tubules [45].

Zhang et al. [44] studied ocean fouling in submerged polysiloxane and PTFE surfaces. They reported that the less hydrophobic surfaces got fouled within a day, while the superhydrophobic surfaces took over three weeks to get fouled. Superhydrophobic or highly hydrophobic surfaces are found to better reduce the interaction between the feed solution and membrane surface. One of the natural examples for superhydrophobic surface is a lotus leaf. The electron microscope image of the lotus leaf shows a waxy coating of low surface energy materials on a high roughness, micro-nano hierarchical structured material (Figure 7). By mimicking this, the researchers could create a superhydrophobic surface.

**Figure 8:**
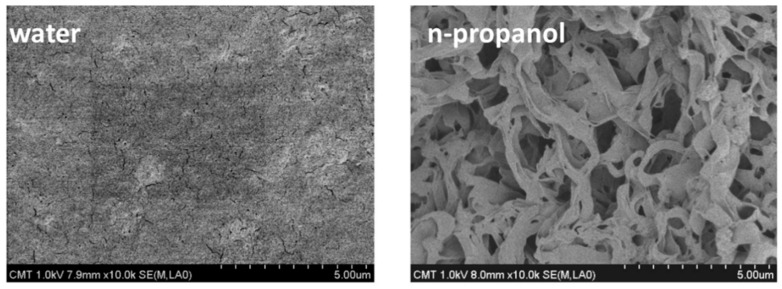
SEM photographs of PVDF membranes prepared by immersing in a water bath and n-propanol bath for 2 s and then immersing into a water bath. Reprinted from [46]. Copyright (2008) with permission from Elsevier.

The roughness on the membrane surface can be created by delayed phase separation in a coagulating bath. This is usually done by the introduction of a weak non-solvent additive in the polymer solution (dope) or in the coagulation bath. Kuo et al. [46] prepared a highly hydrophobic membrane by passing the PVDF/N-methyl-2-pyrrolidone (NMP) casting film through different types of simple alcohols (methanol, ethanol, n-propanol, n-butanol) for 2 seconds followed by immersion into a water bath to achieve the delayed precipitation. They found that compared to the single water bath, the dual bath (alcohol followed by water) produced a rough membrane surface due to the delayed precipitation (Figure 8). The obtained PVDF flat sheet membrane exhibited more porosity and water contact angle of ~140°.

Popular ways to obtain membranes with high or superhydrophobic surfaces can be broadly classified into two types: (i) in situ addition of hydrophobic additives and (ii) ex situ hydrophobic modification of the surface using chemical or plasma treatment. In the in situ method, the polymer dope solution is blended with low surface energy materials such as PTFE [47], PDMS [48], and fluorinated silica (FSi) particles [49], prior to membrane preparation. For instance, Edwie et al. [49] synthesized FSi particles and dispersed them in methanol and NMP. PVDF was then added to prepare the outer doping solution for the fabrication of PVDF hollow fiber membrane. They obtained a water contact angle maximum of ~140° for the FSi-incorporated PVDF membrane. Surface-modifying macromolecules (SMM) have also been used as an additive to enhance hydrophobicity [50,51,52]. The SMMs are oligomeric fluorpolymers with fluorinated end groups. Prince et al. [52] synthesized SMM using the solution polymerization method and blended them with PVDF dope solution to prepare a flat sheet membrane. They were able to obtain a superhydrophobic membrane surface with a water contact angle of 151°. However, the drawback of these in situ methods is that the addition of these particles reduces the porosity of the membranes.

Conversely, in the ex situ method, the membranes are initially prepared and their surface is modified by coating/grafting with low surface energy materials or they are subjected to plasma treatment. Zheng et al. [53] designed lotus-leaf-like superhydrophobic PVDF film through alkali treatment and surface modification. The PVDF film was initially exposed to concentrated sodium hydroxide solution followed by grafting with a mixture of dimethyldichlorosilane (DTS) and methyltrichlorosilane (MTS). The alkali treatment creates micro-nano structures on the surface, while DTS/MTS grafting provides superhydrophobicity. The modified surface resembled structures similar to the lotus leaf with a contact angle of 157°. The same technique could be used to prepare membranes with a superhydrophobic surface.

**Figure 9:**
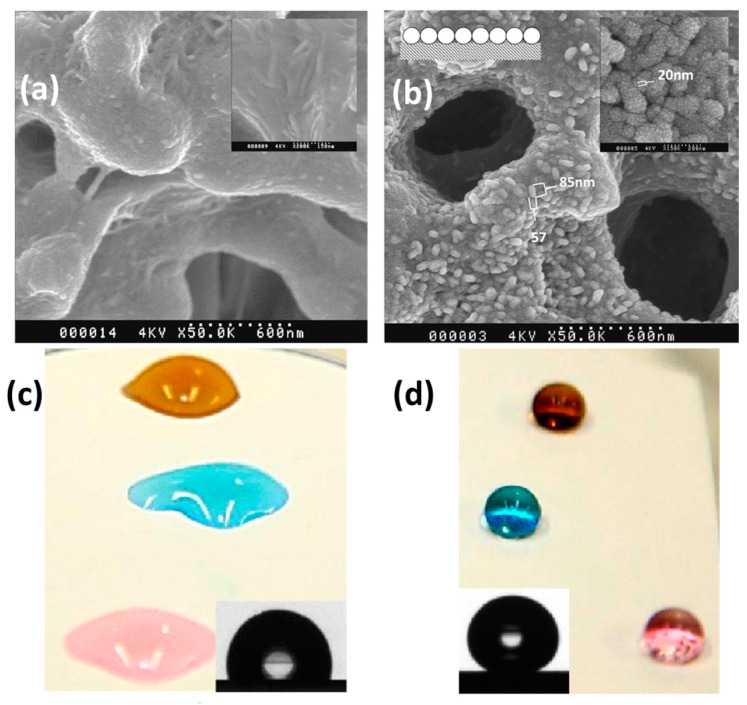
Effect of superhydrophobic modification on PVDF membranes: (**a**) virgin PVDF, (**b**) FTCS–TiO_2_–PVDF membrane (insets at the top right corner of (**a**) and (**b**) are the SEM images at high resolution and at the top left corner of (**b**) is the schematic side view of clusters assumed in a series of cylinders aligned horizontally). Wettability of PVDF membranes (**c**) before and (**d**) after modification with different aqueous solutions (insets: corresponding water contact angle image). Reprinted from [54]. Copyright (2012) with permission from Elsevier.

It is well known that polymeric nanocomposites prepared by incorporating nanoparticles into polymer have additional mechanical strength and roughness. Consequently, nanocomposite membranes can be prepared with superhydrophobicity and good mechanical strength. Razmjou et al. [54] reported superhydrophobic PVDF membranes by the incorporation of TiO_2_ nanoparticles in PVDF membrane surface via in situ blending method followed by modification with 1H,1H,2H,2H-perfluorododecyl-trichlorosilane. The obtained TiO_2_–PVDF nanocomposite membrane displayed superhydrophobicity with an excellent water contact angle of 163° (Figure 9). Hou et al. [55] investigated the effect of hydrophobic CaCO_3_ nanoparticles on the performance of PVDF membrane distillation and found that the contact angle increased to ~124° with the increase in the hydrophobic CaCO_3_ nanoparticles addition.

Lee et al. [56] reported near a superhydrophobic PVDF-co-hexafluoropropylene (PVDF-co-HFP) membrane by in situ incorporation of TiO_2_ functionalized with 1H,1H,2H,2H-perfluoro-octyl-triethoxysilane. The prepared membrane exhibited a contact angle of 149°. In another development, Tijing et al. [57] incorporated carbon nanotube (CNT) as nanofillers to impart an additional hydrophobic property and mechanical properties into PVDF-co-HFP. The contact angle of the prepared membrane was as high as 158.5°. Kujawski et al. [58] worked on the surface modification of ceramic membranes using 1H,1H,2H,2H-perfluorooctyltriethoxysilane and 1H,1H,2H,2H-perfluorotetradecyl-triethoxysilane and they were able to enhance the contact angle from 40° to 148°.

Rastegarpanah et al. [32] prepared a superhydrophobic PES membrane for MD with CA of 150° by grafting trimethylchlorosilane (TMSCl) on tetraethylorthosilicate (TEOS)-treated PES membrane. They observed that increasing the TMSCl/TEOS ratio increases the hydrophobicity of the membrane. Liu et al. [59] modified PES membranes with a dispersion containing hydrophobic modified PES co-polymers. 1H,1H,2H,2H-Perfuorodecyl-methacrylate was used as the monomer for PES hydrophobization. Then, the FMA-C8 grafted PES copolymer was sprayed onto the pristine PES membrane for MD desalination. This modification leads to increased hydrophobicity with the CA = 114° and reduction of maximum pore size, which helps the membrane to improve resistance to pore wetting.

**Figure 10:**
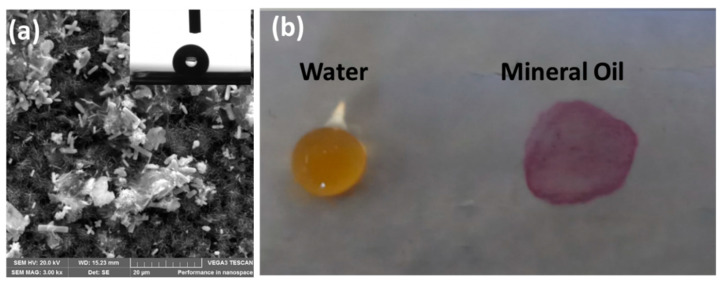
(**a**) SEM image of ZnO rods stearic-acid-modified PES membrane. Inset is corresponding water contact angle image. (**b**) Superhydrophobic and superoleophilic nature of corresponding membrane. Reprinted from [61]. Copyright (2020) with permission from Elsevier.

Recently, Wang et al. [60] prepared a ZnO nanorod array modified superhydrophobic PVDF membrane using an ex situ method. Briefly, ZnO nanorods were grown on the surface of PVDF membrane using a hydrothermal method, which was then modified with 1H,1H,2H,2H-perfluorodecyltriethoxysilane to induce superhydrophobicity. They obtained a water contact angle of 152°. Following this method, Parani and Oluwafemi [61] produced a PES-ZnO composite superhydrophobic membrane where ZnO rods were grown on PES membrane surface and then modified with stearic acid. The resultant water contact angle was 163°. Compared to fluoro silanes, stearic acid is cheaper and more eco-friendly and can be used to induce superhydrophobicity. The obtained membrane was not only superhydrophobic but also superoleophilic, which is useful for oil–water separation applications (Figure 10).

**Figure 11:**
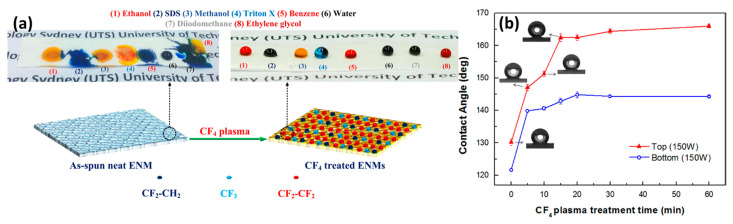
(**a**) Schematic illustrations of the neat and CF_4_ plasma-modified electrospun nanofiber membranes. Reprinted from [66]. Copyright (2017) with permission from Elsevier. (**b**) Water contact angles of top/bottom PVDF membrane surface as a function of CF_4_ plasma treatment time. Reprinted from [64]. Copyright (2014) with permission from Elsevier.

Plasma treatment is a powerful way to modify membrane surfaces. Tian et al. [62] prepared PSf membrane and treated it with CF_4_ plasma for 30 min. The modified membrane exhibited a contact angle of 144°. Wei et al. [63] changed the hydrophilic PES flat sheet and hollow membrane to hydrophobic through CF_4_ plasma treatment. Their results revealed that the plasma modification converted hydrophilic membranes of a contact angle 0° into hydrophobic ones with a contact angle above 120°. Similarly, Yang et al. [64] were able to obtain a superhydrophobic PVDF membrane exhibiting a contact angle of 162.4° by CF_4_ plasma treatment. Pedram et al. [65] designed thin films of fluorocarbon which were deposited on PES membranes through argon plasma sputtering of PTFE targeted in RF magnetron plasma reactor. Recently, Woo et al. [66] prepared PVDF membrane via electrospinning and treated the surface with CF_4_ plasma. The CF_4_-treated electrospun membrane exhibited both superhydrophobicity (CA = ~160°) and superoleophobicity (CA = ~150°), rejecting both water and oil (Figure 11a). Factors such as the influence of RF power, treatment time, gas pressure, and target substrate distance usually play a major role in obtaining the superhydrophobicity of the membrane (Figure 11b).

## 7. Membrane Fouling

Membrane fouling is one of the major issues that deteriorates the long-term performance of MD, such as in other membrane-based separation processes. Fouling can be defined as the accumulation of unwanted materials on the membrane surface or inside its pores [67] which could have detrimental effects, leading to decreasing the efficiency of separation, flux, or even total membrane damage [67,68]. Especially, fouling inside the pores will lead to partial or complete pore blocking or wetting and deteriorate the membrane separation process. The fouling is caused by the aggregation of foulants followed by deposition on the membrane surface or by the direct deposition of foulants on the membrane surface due to surface–foulant interaction. This depends on several factors such as the type of foulant, surface properties of membrane, operating conditions, and the feed water characteristics. 

Membrane fouling is majorly classified into three categories, namely inorganic, organic, and biofouling. Inorganic fouling, usually referred as scaling, is caused by the deposition of inorganic minerals precipitated from the supersaturated feed solution. CaCO_3_ is the most common scale found to form on membrane surfaces due to its low solubility product [69]. Increasing the feed water temperature and alkaline conditions will increase CaCO_3_ scaling due to the decomposition of HCO_3_^−^ ions and their reaction with Ca^2+^ ions present in the feed water. Apart from CaCO_3_, other scales of inorganic minerals include CaSO_4_, MgSO_4_, silica, and Ca_3_(PO_4_)_2_ [70]. Inorganic fouling can be prevented by the addition of chelating agents in the feed water, acidification, decreasing the feed water temperature, etc. The scale that has already formed in the membrane is also removable by washing the membrane with dilute HCl. Gryta et al. [22] demonstrated that simply rinsing the membrane with 3% HCl removes the scales without significant change in the flux and salt rejection. Organic fouling is caused by the deposition of organic materials such as humic acid, proteins, or polysaccharides. The most common foulant is humic acid, a group of organic molecules with aliphatic/aromatic carboxylic/phenolic groups. Humic acid is abundant in natural organic matter (NOM), and is usually found to form scales in membranes through electrostatic and hydrophobic interactions [71]. NOM scale can be removed by rinsing the membrane with dilute NaOH, citric acid, etc. [71,72]. On the other hand, biofouling is due to the growth of biological species, mainly microorganisms in the membrane [73]. Compared to pressure-driven membrane operations such as RO or NF, biofouling is less likely to form in MD membranes due to high salinity and temperature operating conditions. However, certain microorganisms, such as anaerobic bacteria or fungi can still grow and reproduce at high temperatures, causing the biofilm [74,75,76]. Organic fouling formed by extracellular polymer substances can often lead to biofouling as it provides enough nutrients (proteins, dead organisms, nucleic acid, etc.) for the growth of microorganisms. This could cause partial or complete blocking of pores, affecting the rejection rate and flux, and which is sometimes difficult to remove. Biofouling can be reduced by periodic cleaning of membranes, pretreatment of feed water solution, etc. [77]. Recently, Nthunya et al. [78] incorporated functionalized MWCNT/Ag NPs on superhydrophobic PVDF nanofibre membranes for desalination using DCMD. The presence of Ag NPs on the membrane was found to inhibit the formation of biofilm. 

In real-time desalination, fouling is a complex process, and a combination of inorganic, organic, and biofouling can occur. The inorganic and organic fouling can provide a site for microbial growth. Once the biofilm has occurred, it attracts more inorganic and organic matters, and the process can be continuous. In such cases, the fouling is more intense and leads to permanent damage of the membrane. Hence, proper pretreatment of feed water and optimizing the membrane characteristics and operating conditions are necessary to mitigate the fouling in MD applications. For example, the permeate flux (measured in Kg m^−2^ h^−1^ or L m^−2^ h^−1^) is directly proportional to the porosity and is inversely proportional to membrane thickness and dissolved salt in the feed solution. A salt concentration lower than 4.5 wt% is reported to be safe from fouling and scaling [79]. It is anticipated that the hydrophobic membranes may cause severe temperature polarization which thereby affects the evaporation efficiency in the membrane distillation process due to high thermal conductivity. Hence, the development of a dual-layer membrane consisting of hydrophobic and hydrophilic supports may solve this issue. In such cases, the dual-layer membrane is fabricated with a thin hydrophobic active layer supported by a hydrophilic bottom layer with moderate thickness [80,81]. Another factor is the entrapment of air inside the pores, which can affect the transport of water vapor. To overcome this, it is better to deaerate the feed. The deaeration can cause increased permeate flux which could be due to the reduction in a transmembrane temperature [82].

## 8. Membrane Distillation Experiment

**Figure 12:**
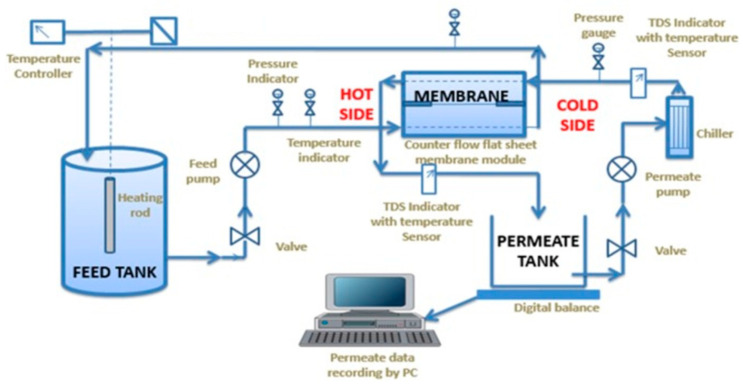
A typical DCMD apparatus. Reprinted from [83]. Copyright (2017) with permission from Elsevier.

A typical DCMD setup widely investigated for desalination of saline water is shown in Figure 12. The setup contains a holder to fix the membrane (flat sheet or fiber) inside. A heater and chiller are included for heating the feed water to be treated and condensing the permeate water vapor, respectively. The feedwater is usually heated to around 40 °C to 80 °C and circulated through the hydrophobic side of the membrane. The cold water at 20 °C to 40 °C is circulated on the other side of the membrane to condense the water vapor permeated from the membrane pores. Flow rate, temperature, and pressure of both feed and permeate water are the important parameters that affect the desalination and need to be monitored during the process. This DCMD design can be replaced with other modules such as VMD, AGMD, or SGMD, provided with their basic requirements (vacuum, airgap, sweep gas) and integrated with other desalination, crystallization, and power generation technologies, which are discussed in the following sections.

The conductivity or total dissolved solids of both feed and permeate water quality is checked to measure the amount of rejection of solutes from the feed water during the process. A laboratory study (for desalination) is usually conducted with NaCl saline solution as a feed. The salt rejection will be calculated following Equation (1).

(1) $$R\;\left(\%\right)=\frac{C_{f}-C_{p}}{C_{f}}*100$$

where *C_f_* and *C_p_* denote the sodium chloride concentration of feed solution and permeate, respectively.

## 9. Applications

### 9.1. Using Standalone MD Configurations

**Figure 13:**
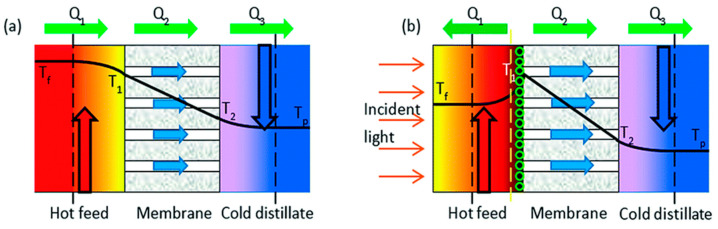
Schematic of (**a**) conventional DCMD and (**b**) novel photothermal DCMD with a nanomaterial coating and localized heating at the membrane surface. Reproduced from [86] with permission from The Royal Society of Chemistry.

Membrane distillation is widely investigated in the treatment of water with a particular focus on desalination. Boubakri et al. [84] studied the capability of DCMD to desalt sea and brackish water with flat sheet polypropylene membranes. The membrane was able to desalt the brackish water without any pretreatment. In the case of seawater treatment by DCMD, due to its high salinity, which causes membrane fouling, they recommended the pretreatment step such as the use of a cartridge filter. DCMD is inefficient for seawater desalination due to its susceptibility to fouling, and, therefore, pretreatment is always recommended. Recently, Jeong et al. [85] applied flocculation and sedimentation pretreatment methods to reduce the organic matter fouling and treated the sewage effluent using DCMD for potable water reuse. Wu et al. [86] set up a solar membrane distillation (Figure 13) using photothermal nanocomposite membranes. In order to achieve high solar absorption, they modified the PVDF membrane with carbon black nanoparticles or silica-coated gold nanoparticles. The modified membrane showed an increase of up to 33% in distillate flux in DCMD mode when the membrane was irradiated with one unit of sunlight.

Hubadillah et al. [87] fabricated a low-cost kaolin hollow fiber ceramic membrane modified with 1H,1H,2H,2H-perfluorodecyltriethoxysilane and reported the removal of arsenic from aqueous solution with 100% removal efficiency using DCMD. Sivakumar et al. [88] proposed VMD for treating mine effluent, which contains a mixture of different ions such as Ca, Mg, Fe, and Al with the TDS of 2332 ppm. The result showed that they were able to desalt the water with 99.9% salt rejection. Recently, Aloulou et al. [89] reported the modification of ceramic sand membrane using long chain fluorosilane grafting and investigated their use for the treatment of oily wastewater using AGMD. The modified ceramic membranes displayed excellent permeate flux (100–150 L m^−2^ h^−1^) with oil rejection (99%) higher than microfiltration and ultrafiltration membrane. In another work, Mousavi et al. [90] prepared polyetherimide (PEI) hollow fiber membranes through an electrospinning process and modified PDMS using dip coating method which was then used for the removal of dye (methylene blue) from wastewater using SGMD. The PDMS-modified PEI membrane showed excellent rejection (99.9%) with antifouling properties.

Zakrzewaska et al. [91] employed MD to concentrate the radioactive substances for subsequent conditioning and disposal. Since there was no adsorption of metal ions on membrane surface and pores, which usually occur with RO membrane, the contamination of radioactive substances and the generation of secondary wastage was minimized. Zakrzewaska et al. [92] also performed the separation of H_2_O/HDO and ^16^H_2_O/^18^H_2_O using the diffusion isotope effect. Wen et al. [93] studied the removal of nuclides containing a mixture of Co (II), Sr (II), Cs (I), and boron from the highly saline radioactive wastewater by DCMD with PP membrane. They reported that boron was effectively removed with the rejection factor of 99.97% even when the feed boron concentration reached 5000 ppm. Recently, Nie et al. [94] reported the decontamination of uranium-contaminated low-level radioactive wastewater using VMD with PTFE membrane. The permeate flux of 11.3 L∙m^−2^∙h^−1^ with extraordinary rejection (>99.99%) against uranium was achieved with simulated wastewater containing (UO_2_(NO_3_)_2_). In another work, Alkhudhiri et al. [95] reported the removal of heavy metal ions (As, Pb, Hg) using AGMD, with good rejection efficiency (96%). They also observed no significant influence of pH of feed water on the efficiency due to the PTFE membrane used.

Koeman-Stein et al. [96] studied the potential of membrane distillation for desalination of cooling tower blowdown (CTBD) water using PTFE membrane. They achieved a concentration of CTBD water by a factor of 4.5 with a water recovery of 78% available for reuse.

The wastewater produced from the oil and gas industries is referred to as oil-produced water, which contains a mixture of various organic and inorganic fractions, including dissolved and dispersed oil compounds. The use of reverse osmosis, microfiltration and nanofiltration membrane technologies in the treatment of the produced water are limited due to their inability to remove these small molecules. Macedonio et al. [97] applied DCMD using PVDF and PP membrane for the treatment of produced water with an overall salt rejection of 99% and total carbon rejection higher than 90%. Mokhtar et al. [98] applied DCMD with PVDF membrane to treat the effluent from the rubber industry and were able to achieve 96% removal efficiency. In another experiment, Mokhtar et al. [99] reported the use of MD for the removal of dye from the aqueous solution using reactive black (RB5) dye as a model dye. The dye rejection obtained was 99.82%, though the permeate water flux was found to be low. MD has also been utilized for the concentration of food products. Hausmann et al. [100] investigated DCMD performance in the processing of skim milk and whey using PTFE membrane. They concluded that MD has potential in concentrating skim milk and whey as it showed high rejection (>99%) for all dairy components.

### 9.2. Using Hybrid Membrane Distillation

The MD process is flexible and can be integrated with other processes to set up a hybrid MD which minimizes the limitations of individual process and significantly contributes to the process intensification. The advantages of hybrid-MD are multifold such as enhanced treatment efficiency, high resource recovery, and reduction in the operation cost.

A system that couples photocatalysis and membrane processes is called a photocatalytic membrane reactor (PMR). This is advantageous over conventional photoreactors because of the possible recovery of photocatalyst when a suspension of photocatalyst is applied. Mozia et al. [101] investigated PMR using DCMD for the degradation of azo dyes (Acid Red 18, Acid Yellow 36 and Direct Green 99). They suggested the use of MD membrane over RO membrane for PMR since the latter is a pressure-driven membrane where significant fouling was observed. Phattaranawik et al. [102] investigated a hybrid process incorporating membrane distillation in a submerged membrane bioreactor (MBR) to separate suspended solids from the effluent. They called the integration setup a membrane distillation bioreactor (MDBR). A flux decline in MDBR was observed compared to mere MBR, however, the permeate quality was improved.

**Figure 14:**
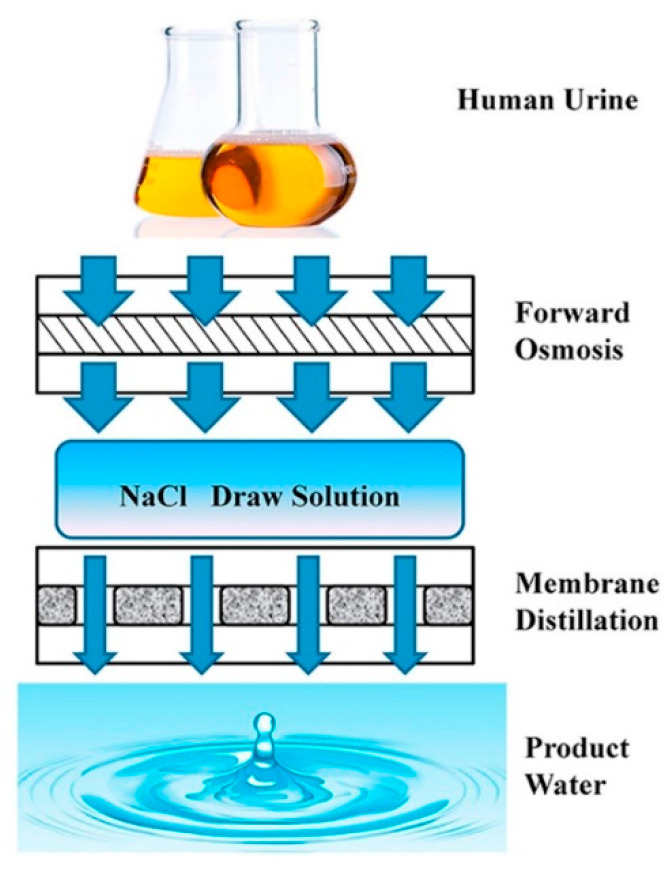
Schematic diagram for the integrated forward osmosis-membrane distillation process for human urine treatment. Reprinted from [104] Copyright (2016) with permission from Elsevier.

Quist-Jensen et al. [103] coupled ultrafiltration (UF)/DCMD for the concentration of orange juice. The raw juice was previously clarified by UF in order to remove suspended solids and juice turbidity, which was concentrated from 9.5 °Brix to 65 °Brix through a two-step DCMD process. Liu et al. [104] investigated the integrated forward osmosis (FO) and MD process to recover water from human urine (Figure 14). They adopted NaCl solutions at different concentrations as draw solutions in FO processes, which were also the feed solutions for the subsequent MD process. The result showed that the product water of FO-MD had much higher quality than that of MD alone.

**Figure 15:**
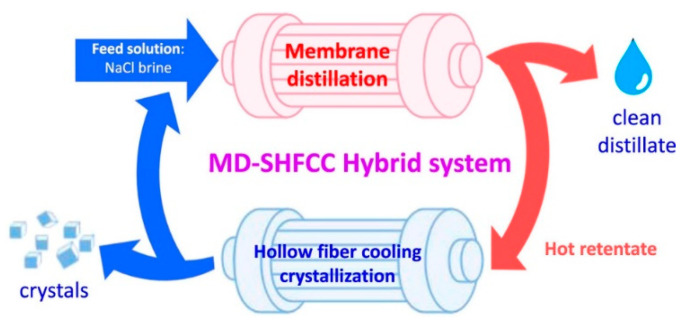
Concept of the MD–SHFCC desalination system. Reprinted from [106]. Copyright (2018) with permission from Elsevier.

**Figure 16:**
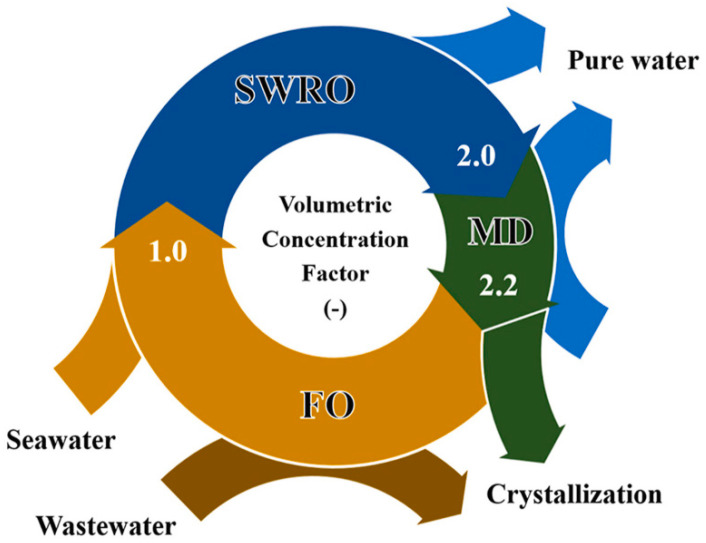
Concept of circular brine reclamation using SWRO, MD, and FO. Reprinted from [108]. Copyright (2021) with permission from Elsevier.

Seawater reverse osmosis (SWRO) plants have been installed globally to produce drinking water. The integration of MD with SWRO could enhance the water recovery with the reduced cost of water (COW). Bindels et al. [105] studied several hybrid systems of RO-MD, RO-UF-MD, and RO-NF-MD and evaluated their water recovery. They observed that compared to other hybrid systems, a simple RO-MD hybrid system with the anti-scalant produced water recovery as high as 84.5% at low COW. The use of MD in the MD-RO hybrid system can also be considered for the achievement of zero liquid discharge. Industries traditionally use crystallizer or evaporators to treat highly concentrated brines to recover the salt. However, these techniques consume a lot of energy and are not cost-effective. Membrane crystallization appears as an emerging, less energy-consuming technology to recover salts from the concentrated brine. Recently, Luo et al. [106] designed the hybrid MD–solid hollow fiber cooling crystallization system (Figure 15) to simultaneously produce salts and crystals. The reported hybrid system simultaneously produced high purity distillate with good MD water flux and NaCl salt crystals with a yield of 64 g/kg feed. The membrane distillation–crystallization has also been developed to recover other valuable organic and inorganic resources such as ammonia, heavy metals, and proteins [107]. In another work, Son et al. [108] developed a hybrid system involving SWRO- MD–FO (Figure 16) in order to achieve zero liquid charge by circular brine reclamation. In this system, the SWRO brine was heated before entering the MD process as a feed and the concentrated brine from the MD outlet served as the draw solution for FO. The highly concentrated draw solution was diluted by wastewater through FO permeation to reach the typical seawater salinity. The obtained effluent was blended with SWRO feed and the brine discharge was avoided.

**Figure 17:**
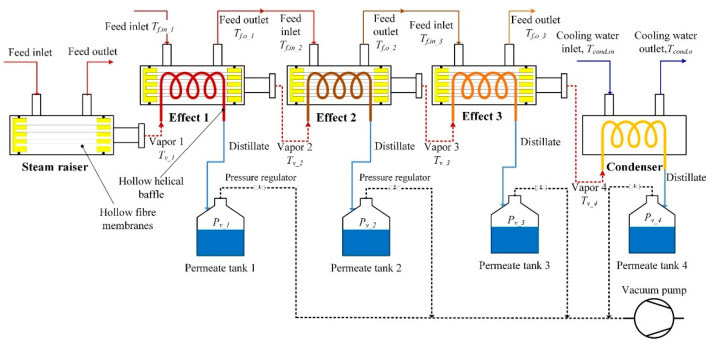
Schematic diagram of a three-effect HF V-MEMD system (the solid lines illustrate the liquid, while the dashed lines denote the vapor). Reprinted from [112] Copyright (2020) with permission from Elsevier.

Multi-effect distillation (MED) is the oldest process and has been employed in various industries, especially in the desalination industry [109,110]. MED is the advance of single effect distillation whereby a series of single effect distillation systems is used to prevent the large amount of heat energy rejected to the surroundings. In MED first stage, steam is applied to evaporate sea (saline) water. Instead of condensing the vapors (produced from the hot seawater) directly as in single effect distillation system, the evaporated hot vapor in the first stage is sent to the second stage and used to evaporate the (fresh) seawater, and the process can be repeated. The condensation of steam water and evaporated saline water occurs in each effect and can be collected together as pure distillate while the brine gets concentrated in sequence of effect and collected. Vacuum can be applied for the fast and efficient collection of the water vapors. Understanding the temperature drop in each effect and knowing that MD can operate at low temperatures, MD can be integrated with MED for the effective utilization of thermal energy for desalination. In the integrated set up, called multi-effect membrane distillation (MEMD), the heat associated with the vapor condensation in each effect is used to heat the feed water and passed through the MD process where lower temperature and pressure conditions exist, and thermal energy is effectively used for distillation. This is characterized by the term gained output ratio (GOR) [111] which refers to the ratio of recoverable heat (latent heat of evaporation) over the total heat supplied to the system. Increases in the number of effects usually increases GOR, however there will be a reduction in the permeate flux when a large number of effects are used. Three to four effects are usually recommended to achieve high permeate flux and GOR. For instance, Li et al. [112] recently developed hollow fiber vacuum multi-effect membrane distillation (V-MEMD) (Figure 17) for the treatment of brine treatment with the concentration of >200 g/L. This integrated model consists of three effects and is reported to reduce the energy consumption by ~60% compared to its single effect design, and achieved permeate flux of ~5 Kg m^−2^h^−1^ for three effects with the GOR ~2.6.

**Figure 18:**
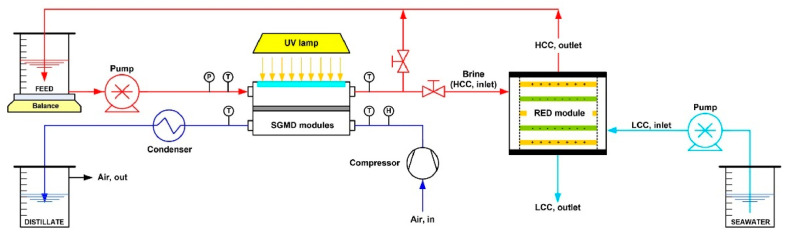
Scheme of the lab-scale photothermal SGMD-RED setup. Reprinted from [114] Copyright (2020) with permission from Elsevier.

Reverse electrodialysis (RED) is a process whereby energy is generated by transport of (salt) ions across the ion exchange membrane due to salinity gradient [113]. As MD results in the production of highly concentrated saline feed, this process can be used to produce a high salinity gradient for power generation using RED. Recently, Avci et al. [114] demonstrated a solar MD–RED hybrid process for the light–heat–energy conversion (Figure 18). They used a photothermal (Ag nanoparticle-incorporated PVDF) membrane which was able to generate heat by ultraviolet irradiation. The heat produced was then used for heating the feed in SGMD. The cold distillate was collected separately while the concentrated brine from the feed side was sent to high-concentration component (HCC) of the RED module. The low salinity component (LCC) was fed with the low saline water. The concentration gradient between HCC and LCC solutions drives the positively and negatively charged (salt) ions in opposite directions across the ion exchange membranes generating the ionic current. This hybrid set up is a demonstration for the process intensification in desalination with a recovery ratio (87.5%) higher than individual RO process and which can simultaneously produce freshwater through MD and power generation (maximum power density for membrane pair, MP, reported 0.9 W/m^2^) through RED. In another work, Tufa et al. [115] demonstrated an integrated SWRO–MD–RED system and was able to produce RED power density of 2.2 W/m^2^ MP. Compared to individual SWRO, the integrated system exhibited 23% reduction in the energy consumption in the desalination.

## 10. Membrane Distillation Pilot Plants

**Figure 19:**
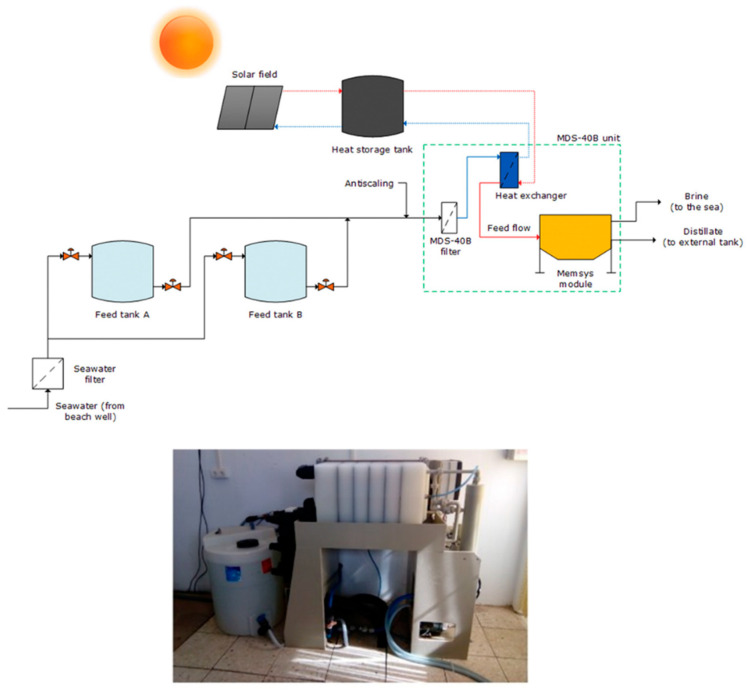
Diagram showing the main components of the seawater desalination pilot plant and Aquaver’s prototype. Reprinted from [117]. Copyright (2020) with permission from Elsevier.

Currently, desalination technology majorly depends on RO membranes. However, over the past two decades, MD technology is emerging in the field of desalination. This has pushed the research towards full-scale/pilot plant studies of MD and a limited number of publications are available for outdoor MD testing with the major focus on desalination. Several manufacturers [111,116] who have installed pilot plants include Fraunhofer ISE, Memsys, Mediras, SolarSpring from Germany, Keppel Seghers from Singapore, and Aquastill from the Netherlands. They are mainly located in Germany, the Netherlands, USA, Saudi Arabia, Singapore, and Qatar and they are contributing about 2% to the share of total desalination. The membrane material that is mostly used is PTFE due to its excellent chemical and thermal stability, although a few pilot plant studies employ PE membranes. The most common configurations in these pilot plants are AGMD, PGAMD, and V-MEMD. The spiral wound, flat sheet, and hollow fibre membranes are employed with the active area ranging from ~1.8 to 168 m^2^. Although the rejection rate is excellent (99%), the permeate flux and GOR are reportedly low. For instance, Andres-Manas et al. [117] reported the assessment of pilot plant system for seawater desalination based on V-MEMD developed by Memsys (assembled by Aquaver BV) installed at the University of Almeria (Figure 19), 150 m away from the seashore. A maximum of 8.5 L m^−2^ h^−1^ distillate flux was obtained and the maximum GOR was 3.19. In another work, Andres-Manas et al. [12] reported the assessment of a V-AGMD pilot plant developed by Aquastill, installed in Plataforma Solar de Almería, Spain for sea water desalination. This plant was reported to produce 8.7 L m^−2^ h^−1^ distillate flux with 13.5 GOR.

## 11. Conclusions and Future Outlooks

In conclusion, the principles of MD were reviewed and the conventional configurations of MD along with recent designs were discussed in this review. An overview of membrane materials, including a variety of polymers and ceramic materials and their method of fabrication, were further discussed. Though there are many polymers used, PVDF is still a successful and popular membrane material to prepare MD membranes at lab-scale, and among the different fabrication methods, the phase separation methods, majorly NIPS method, are still popular at lab-scale and large-scale levels. Compared to neat membranes, nanocomposite membranes offer great advantages by increasing the membrane surface roughness. Particularly, TiO_2_, ZnO, and SiO_2_ nanomaterials have been incorporated in the polymer matrices to prepare these nanocomposite membranes, which require further modification with low surface energy molecules for enhanced hydrophobicity/superhydrophobicity. This hydrophobic modification is usually carried out by coating with fluoralkyl silanes or CF_4_ plasma treatment. In general, fabrication of membranes with optimum thickness exhibiting rough and superhydrophobic layers supported by hydrophilic layers with an asymmetric structure is preferable for MD applications. This can yield a higher rejection with the good permeate flux for the long-term operation in membrane distillation. Since the top hydrophobic layer only accounts for separation, it must be thin enough, while the remaining layers can be hydrophilic for higher permeate flux without compromising the rejection efficiency.

The membrane distillation process offers several magnitudes of qualitative advantages over other membrane separation processes. For the past two decades, membrane distillation has been used for a variety of applications such as desalination of brackish water, seawater, and removal of heavy metals, toxic metals, radioactive metals, industrial effluents, which are difficult to do using other separation technologies. Furthermore, concentration of fruit juices and organic volatiles have also been attempted using MD. Although there are numerous reports on MD for different applications conducted at lab-scale/large-scale levels, the permeate flux and the GOR obtained by MD is still lower than that of the conventional RO, and, in addition, most available studies lack information on long-term operation and membrane’s fouling resistance. Commercialization of MD is still at its early stages, and mostly PTFE membranes are used in the pilot-scale/commercial MD applications. While the PTFE membranes meet many requirements, high hydrophobicity, thermal and chemical stability, high cost, and complicated membrane fabrication methods are the major drawbacks for industrial-scale applications. Hence, it is critically important to find low-cost membrane materials and fabrication technologies to produce membranes with high hydrophobicity and high thermal and chemical stability for MD applications. These can be addressed by using cost-effective membrane materials such as polysulfones together with the advancement of nanotechnology. Incorporating nanoparticles on such membrane surfaces followed by superhydrophobic modification with cost-effective hydrophobic modification agents such as stearic acid will be the way to fabricate and commercialize an efficient MD membrane that can be utilized for water treatment processes. In addition, more research should be carried out to utilize renewable solar energy and low-grade heat energy to minimize conventional use of heat sources and maximize the permeate flux. Meanwhile, the integration of MD with other desalination technologies such as RO, FO, MBR, multi-effect desalination, or RED can be considered as MD can operate even at hypersaline concentrated solutions.

## Figures and Tables

**Table 1:** MD conventional configurations and their advantages, disadvantages, and applications.

Configuration	Advantages	Disadvantages	Applications
DCMD	Simple design and operation, high flux	High conductive heat loss, low non-volatile rejection	Desalination of sea water, brackish water, removal of various contaminants
VMD	Negligible heat loss, high flux	Risk of membrane wetting due to high pressure difference	Concentration of fruit juice, inorganic acids, recovery of volatile organic compounds, concentration of RO brine
SGMD	Low conductive heat loss, high flux	Requires large condenser, expensive	Concentration of fruit juice, removal of non-volatile organics, ethanol processing
AGMD	High non-volatile rejection, high purity Low membrane wetting, low heat loss	High mass transfer resistance, low permeate flux	Wastewater produced in water treatment, separation/removal of inorganic acids and organic compounds

## Data Availability

Not applicable.
